# Fully degradable, transparent, and flexible photodetectors using ZnO nanowires and PEDOT:PSS based nanofibres

**DOI:** 10.1038/s41528-025-00385-9

**Published:** 2025-03-10

**Authors:** Xenofon Karagiorgis, Nitheesh M. Nair, Sofia Sandhu, Abhishek Singh Dahiya, Peter J. Skabara, Ravinder Dahiya

**Affiliations:** 1 School of Engineering, University of Glasgow, Glasgow, G12 8QQ UK; 2 School of Chemistry, University of Glasgow, Glasgow, G12 8QQ UK; 3 Institute of Smart Sensors, University of Stuttgart, Stuttgart, 70569 Germany; 4 Bendable Electronics and Sustainable Technologies (BEST) Group, Northeastern University, Boston, MA 02115 USA

## Abstract

Transparent light detection devices are attractive for emerging see-through applications such as augmented reality, smart windows and optical communications using light fidelity (Li-Fi). Herein, we present flexible and transparent photodetectors (PDs) using conductive poly(3,4-ethylenedioxythiophene): polystyrene sulfonate (PEDOT:PSS): Ag nanowires (NWs) based nanofibres and zinc oxide (ZnO) NWs on a transparent and degradable cellulose acetate (CA) substrate. The electrospun (PEDOT:PSS): Ag NW-based nanofibres exhibit a sheet resistance of 11 Ω/sq and optical transmittance of 79% (at 550 nm of wavelength). The PDs comprise of ZnO NWs, as photosensitive materials, bridging the electrode based on conductive nanofibres on CA substrate. The developed PDs exhibit high responsivity (1.10 ×10^6^ A/W) and show excellent stability under dynamic exposure to ultraviolet (UV) light, and on both flat and curved surfaces. The eco-friendly PDs present here can degrade naturally at the end of life – thus offering an electronic waste-free solution for transparent electrodes and flexible optoelectronics applications.

## Introduction

Photodetectors (PDs) are crucial devices for flexible optical electronic systems and their applications in high-capacity wireless communication, light fidelity (Li-Fi) and multiuser interaction^1,2^. The wide span of their application also includes safety systems in the automotive industry, medical diagnostics, logistics, asset tracking, unmanned retail stores and environmental monitoring^3–6^. They also provide attractive alternative solutions for human activity monitoring in a smart home or privacy-sensitive environments without incurring privacy, and latency issues that affect the conventional vision or camera-based approaches^7^. With additional features such as transparency, flexibility and conformability, the PDs also find application in wearable technology and smart textiles^8–14^. As a result a variety of PDs, capable of operating over a wide spectral range, have been reported in literature using inorganic and organic materials and methods^15–23^. Many of them have been developed using conventional microfabrication techniques such as electron beam lithography (EBL)^24,25^, photolithography^26,27^, nanoimprint lithography (NIL)^28,29^, etc. While these methods enable accurate and consistent patterning of PDs, they also come with drawbacks such as high cost, complexity, and limitations in terms of choice of materials^30^. Further, such devices are mostly in a planar (rigid) form factor. To use these devices in daily life applications, it is necessary to have them in a flexible form factor so that they can be integrated into wearable and mobile systems such as flexible displays, wearable photonic textiles, and health montoring^31,32^. One strategy to achieve flexible form factor is to embed or integrate the rigid devices into flexible substrates as hosting matrices using the heterogeneous integration approach^33,34^. For example, we demonstrated in the past how conventional rigid inorganic devices can be integrated on flexible substrates to obtain conformable systems such as electronic skin and smart sensor patches^35–37^. However, under high deformation, the rigid devices cannot maintain their functionality and, because of the large difference in Young’s modulus between the rigid and flexible materials, interface fracture results in the device detachment. To overcome such challenges, the entire optoelectronic system, including the active layer, and the electrodes, must be mechanically flexible and preferably developed using a facile and resource-efficient manufacturing route.

Active electronics on textiles have been explored to address the above shortcomings using either printing functional inks on clothes and fabric or yarn and fibre-based devices^38–40^. The electrospun nanofibre-based approach is another attractive option which has been used to draw nanofibres from a viscoelastic fluid^41^. In addition to being cost-effective, it offers scalability, customisability and the capability to successfully produce nanofibres, with diameters down to tens of nanometers, from a rich variety of materials, including polymers, ceramics, small molecules, and their combinations^42–45^. Further, it is possible to use biodegradable materials such as PEDOT:PSS, polyethylene oxide (PEO), poly-L-lactic acid (PLLA), poly(lactic-co-glycolic acid) (PLGA) and chitosan etc., to obtain devices with features such as transparency and antibacterial properties^46–55^. A wide range of degradable sensors, PDs, energy and electronic devices have been reported in the literature in recent years^38,56–60^. The degradability of electronic devices is an important feature which is desired for future electronic waste-free electronics^14,61^. By combining polymers with materials such as carbon nanotubes (CNTs), 1D/2D materials^62,63^, transition metal dichalcogenides (TMDs) such as MoS_2_ and MoSe_2_ and metal oxides such as NWs of ZnO and TiO_2_ it is possible to obtain nanofibers with enhanced optical and electrical properties^57,64–69^.

Considering the above, we report here fully degradable, transparent, and flexible PDs. The developed PDs consist of transparent conductive electrodes based on PEDOT:PSS/AgNWs electrospun nanofibres, on flexible and biodegradable cellulose acetate (CA) films. With sheet resistance of 11 Ω/sq and optical transmittance of 79% at a wavelength of 550 nm, the electrospun (PEDOT:PSS): AgNW-based nanofibres, offer an attractive eco-friendly solution for transparent conductive electrodes (TCEs). Alternative facile methods such as dip and spray coating of various organic/inorganic composites have been reported in the literature to obtain low sheet resistance and flexible TCEs. But, their reproducibility is a major challenge because of uncontrollable fabrication steps. The TCEs here were patterned using a mechanical blade cutter to develop the two sides of the electrodes for PDs. Following this, the photosensitive material, i.e. ZnO NWs, was drop casted to bridge the two electrodes. The developed PDs showed excellent responsivity (1.10 ×10^6^ A/W). The device demonstrated good stability under dynamic exposure to UV light on both flat and curved surfaces and optical transparency of 70%. Moreover, the full degradability of devices is an attractive solution for emerging challenges such as electronic waste. The obtained results show the presented devices hold considerable potential for flexible and see-through optoelectronics and wearable systems.

## Results

### Fibrous electrodes and ZnO NW integration

**Fig. 1:**
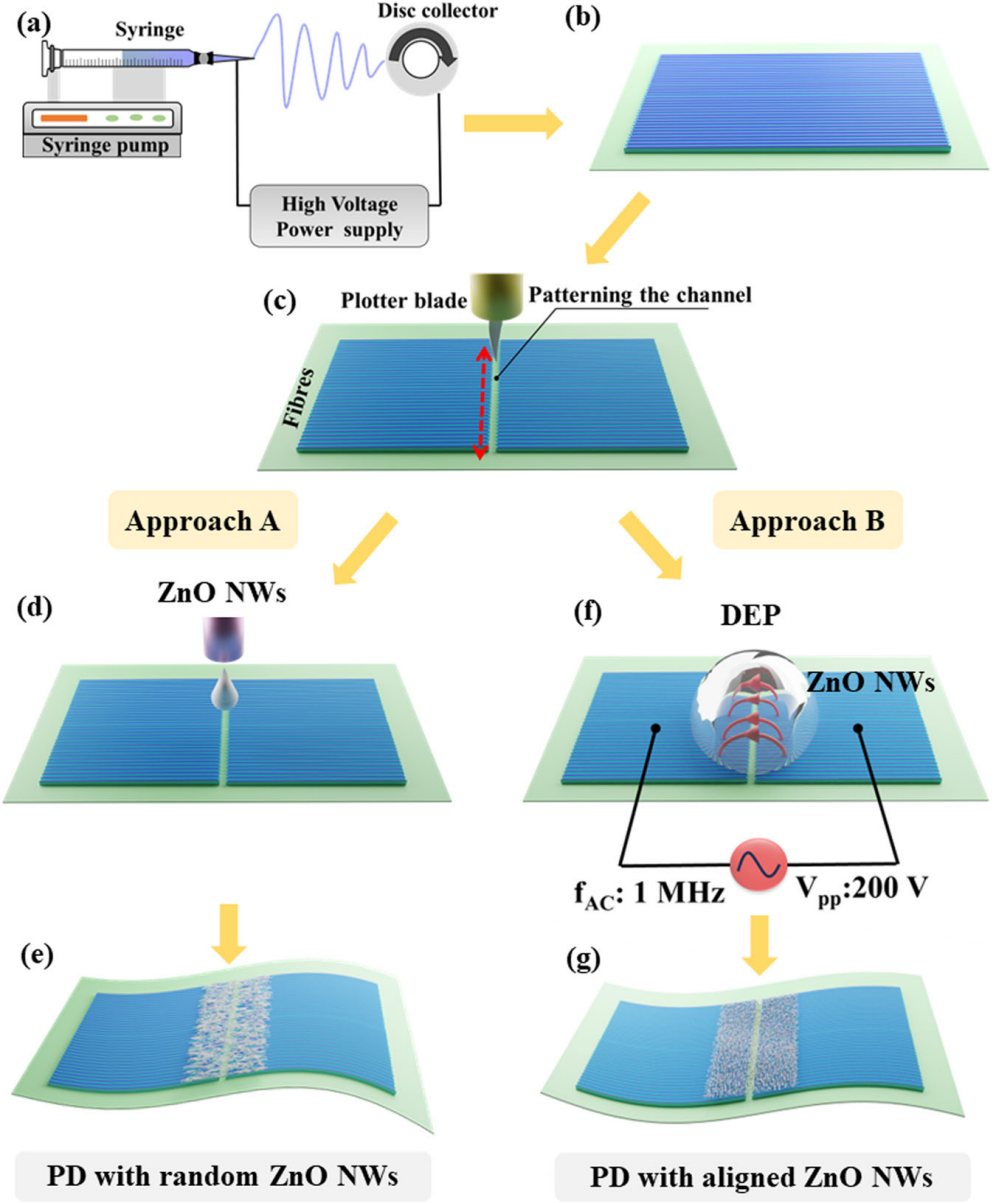
Schematic illustration of the device preparation. **a** Electrospinning setup. **b** Electrospun fibres on the CA substrate. **c** Blade cutting. **d** Drop-casting of ZnO NWs. **e** Device with randomly oriented ZnO NWs dispersion. **f** DEP technique, V_pp_: peak-to-peak voltage and f_AC_: alternate signal frequency. **g** Device with aligned ZnO NWs.

Figure 1a is a schematic of the electrospinning setup used to fabricate the conductive PEDOT:PSS-based fibres. The CA substrate was then prepared by casting the solution onto a petri dish, and the electrospun PEDOT:PSS fibres subsequently deposited on this flexible substrate exhibited a sheet resistance of approximately 11 Ω/sq. Figure 1b shows the electrospun PEDOT:PSS-based fibres on the CA substrate. To create the electrodes for PDs, the CA substrate with PEDOT:PSS fibres was patterned using a blade-cutting machine, as shown in Fig. 1c. Supplementary Fig. 1a illustrates the patterned fibrous electrodes on the biodegradable substrate, with the insert SEM image showing the morphological characteristics of the fibres, which had an average diameter of approximately 400 ± 2.28 nm (from 100 samples). Next, the ZnO NWs were drop-casted between the fibrous electrodes (Fig. 1d) to form the device with randomly oriented NWs (Fig. 1e). Further, the dielectrophoresis (DEP) method (Fig. 1f) was used to orient the ZnO NWs (Fig. 1g) by using the same solution.

To assess the flexibility of the developed devices, we used a butterfly-shaped electrode pattern and powered four LEDs integrated with this pattern. The butterfly-like electrode was bent at various radii, as shown in Supplementary Fig. 1b. As indicated by the images, all four LEDs remained illuminated across all bending diameters. This observation demonstrates the suitability of PEDOT:PSS-based fibres as a promising candidate for TCEs in flexible and transparent electronics. Supplementary Movie 1 shows the outstanding flexibility and durability of developed PEDOT:PSS fibres, which are elegantly patterned to make butterfly-shaped electrodes.

### Fibre-based PD with randomly oriented ZnO NWs

**Fig. 2:**
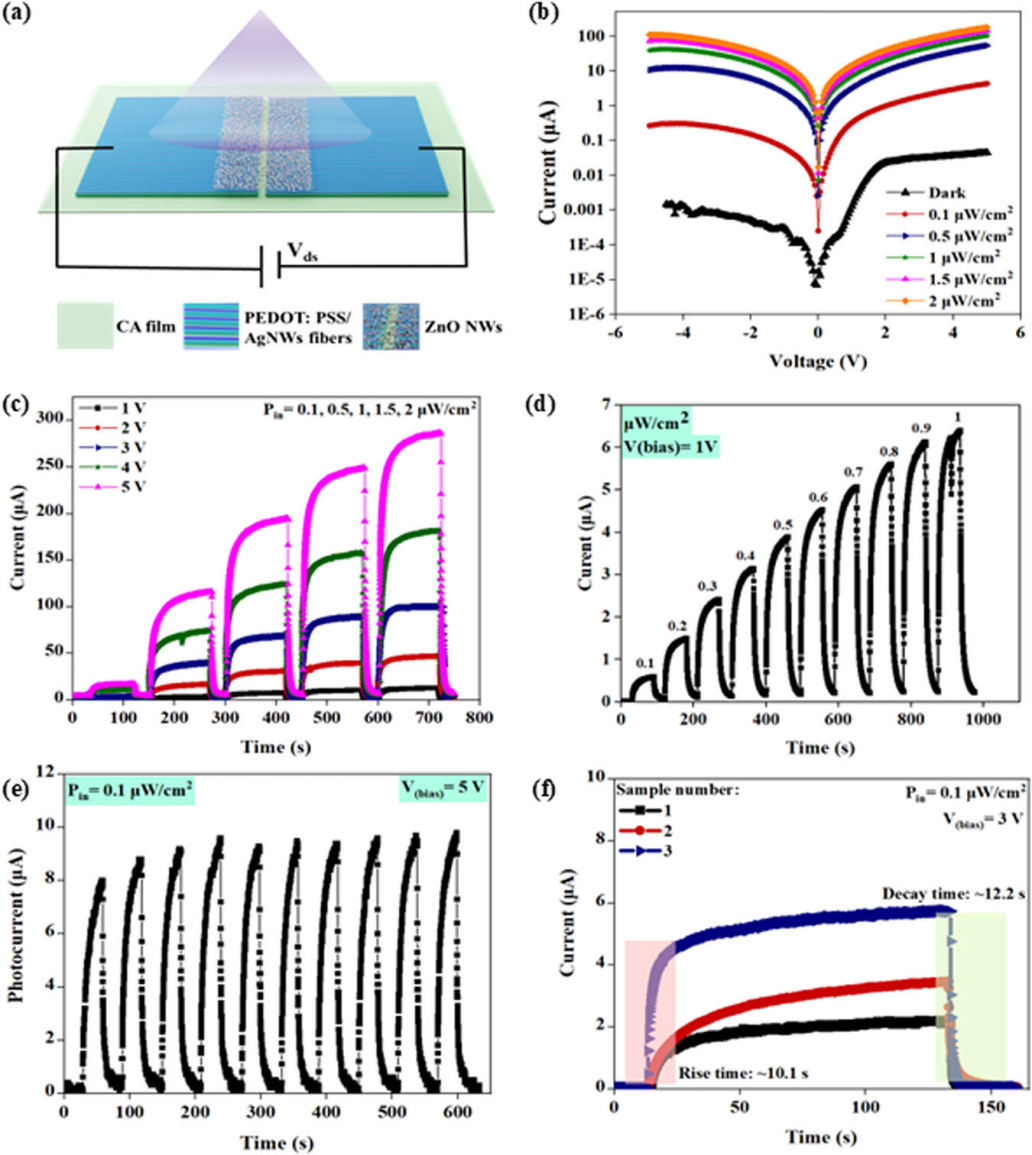
Electrical characterisation of the r-PD. **a** Schematic of the fabricated fibre-based PD. **b** Current vs voltage plot. **c** Light-illuminated temporal response at different bias voltages (1–5 V) under different UV illumination intensities. **d** Stepwise incremental response under different light intensities from 0.1 to 1 μW/cm^2^. **e** Cyclic stability, and **f** repeatability and decay time.

The as-fabricated fibre-based PDs were electrically characterised to evaluate their photoresponse. This involved exposing the PD to UV light generated by an LED with a wavelength of 365 nm, under ambient conditions. The UV light at 365 nm is used due to its significance in environmental and health applications^70,71^. Examples of such substances and phenomena include fluorescent dyes and markers, organic pollutants, environmental contaminants, and biological indicators and pathogens^72–74^. By using UV light at 365 nm, targeted detection, optimisation of sensitivity, validation of applications, and standardisation can be achieved, ultimately leading to improved monitoring, diagnostics, and comprehension of environmental and health-related phenomena. The schematic of the fibre-based PD is shown in Fig. 2a and an SEM image of the device is shown in Supplementary Fig. 2a. Figure 2b illustrates the semi-log current-voltage (I-V) characteristics of the PD under different intensities (0.1-2 μW/cm^2^) at room temperature and ±5 V bias. The obtained results reveal that the PD exhibits behaviour that is consistent with back-to-back symmetrical Schottky contacts. A notable enhancement in the device current was observed with an increase in the UV power intensity, primarily due to the enhanced absorption of photons by the ZnO NWs. The increased absorption of photons led to a higher generation of electron-hole pairs within the ZnO NWs, consequently contributing to the overall photocurrent of the PD.

Systematic investigations were conducted to examine the photoresponse of the fibre-based PD by varying power intensities and bias voltages. Figure 2c illustrates the time-domain photoresponse of the PD under different bias voltages (ranging from 1 to 5 V) and light illumination intensities spanning 0.1 to 2 μW/cm^2^. In all cases, the photocurrent demonstrated an upward trend with increasing incident UV light intensities, attributable to the enhanced generation of electron-hole pairs. For instance, the photocurrent exhibited a substantial rise from 4 nA under dark conditions to 291 μA when exposed to UV light with an intensity of 2 μW/cm^2^ and an applied voltage of 5 V. Figure 2d shows the stepwise response (step of 0.1 μW/cm^2^) of the device under different light intensities ranging from 0.1 to 1 μW/cm^2^, with a fixed bias voltage of 1 V. The reproducibility of the photocurrent obtained from the device was demonstrated by subjecting it to 10 cycles of exposure to a constant light intensity of 0.1 μW/cm^2^ at a bias voltage of 5 V (Fig. 2e). For statistical analysis, three devices were subjected to the same conditions: 0.1 μW/cm^2^ light illumination and a bias voltage of 3 V. The experimental results, shown in Fig. 2f, indicate that all samples have a rise time of approximately 10.1 s and a decay time of about 12.2 seconds. These long response and recovery times are common in ZnO-based photodetectors due to persistent photoconductivity^75,76^. These rise and decay times are well-suited for applications such as UV dosimeters, which track UV exposure to prevent overexposure to sun light, particularly to UVA radiation (315–400 nm)^77^. While sunblock lotions offer protection against UVB, there are limited solutions available for monitoring UVA exposure. The presented photodetectors could be part of the wearable systems that can track long-term UV exposure, especially in the UVA range, to prevent the health risks such as skin aging and cancer. Additionally, the slow responding photodetectors would be better fit for environmental monitoring in applications such as measuring sunlight intensity in agricultural settings, light pollution tracking in urban areas, and solar radiation monitoring for renewable energy systems. Gradual changes are observed in these applications over a period. The flexibility and biodegradability features also make these photodetectors ideal for above applications, as well as in wearable transient technology, offering an eco-friendly, use-and-throw (single-use) option without much ecological footprint.

The primary objective of this study is to address the challenges related to the fabrication of transparent, flexible, and degradable PDs. Future research will explore integrating ZnO NWs with more photosensitive materials to improve device performance further. Variations in the photoresponses of the three samples under the same conditions were also observed: sample 1 showed a current of 2.42 μA, sample 2 showed 3.41 μA, and sample 3 showed 5.75 μA. To improve reproducibility across devices from a single or multiple batches, several factors need to be considered, starting from synthesis of NWs with controlled dimensions, doping and surface states etc. Then consistent deposition of uniform NW-based layers needs to be ensured. In the present case, the drop-casting method used for fabrication of photoactive layer is inherently less precise and could lead to inconsistencies in the density and distribution of NWs. This affects the number of NWs incorporated into each device and incorporation of NW-network in the sensing channel. The disordered arrangement in each sample creates multiple, unpredictable paths for light interaction with the NWs and increases the chances of non-uniformity among devices. The metal-semiconductor (MS) interface is another critical factor that governs the charge transport in the device. Because of the low dimensionality of NWs, it is challenging to realise high-quality or near-ideal MS contacts because of the electrostatics involved at nanocontact interfaces^78^. A combination of above factors could lead to differences in output current with UV exposure. This said, the observed variation in the output current could be compensated using an appropriate conditioning circuit made of transimpedance amplifiers (converts the output into an adequate voltage), ADCs, and microcontroller. Our future work will systematically examine these factors to improve the overall consistency and performance uniformity of photodetectors.

**Fig. 3:**
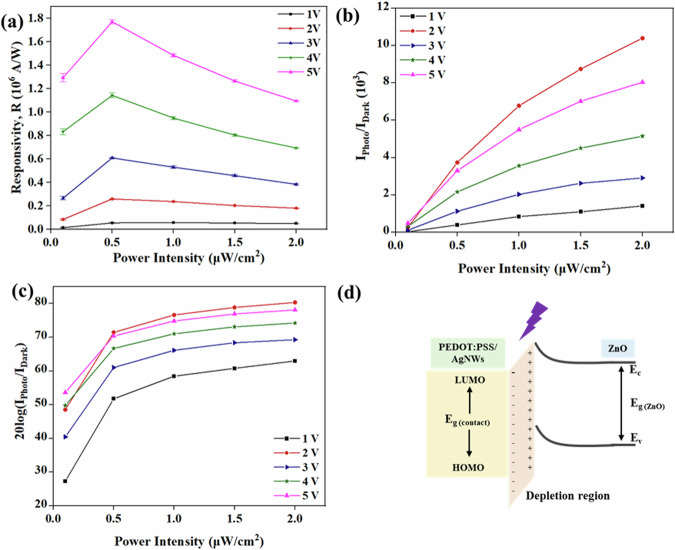
Performance of the r-PD. **a** Responsivity. **b** Current ON/OFF ratio. **c** Linear dynamic response (LDR) plot under different illumination intensities. **d** Schematic of energy level diagram of the PEDOT:PSS-Ag NWs/ZnO heterostructure^87^.

Photodetection response characteristics such as the responsivity (R), current ON-OFF ratio, and the Linear Dependent Range (LDR), were evaluated as depicted in Fig. 3. This analysis is aimed to provide a comprehensive understanding of the performance of the fabricated devices and their photodetection capabilities. The R of a PD is a measure of its sensitivity to incident light, and it is defined as the ratio of the photogenerated current to the illumination power intensity^79^, following Eq. 1:

1 $$R=\frac{{I}_{{Photo}}}{{P}_{{in}}}$$

Where ${I}_{{Photo}}$ is the photogenerated current and ${P}_{{in}}$ is the UV illumination power intensity. Figure 3a plots R under various exposed intensities and bias voltages, with a good value of >1.10 ×10^6^ A/W at 5 V biasing under 0.5 µW/cm^2^ of UV light intensity. The responsivity exhibited an increasing trend with higher biasing voltage, attributed to the enhanced photocarrier separation resulting from stronger electric fields. For each biased voltage, the peak value of R was recorded at around 0.5μW/cm^2^. The value of R decreases with further increase in the light intensity. Such a trend has been observed in previously reported photodetectors as well which is attributed to the hole-trapping saturation^80–82^. The recorded high R values can be attributed to several factors, including the low sheet resistance of the fibrous electrodes and the high surface area and aspect ratio of the wire-like structure of ZnO NWs that allows for more oxygen and water molecules to be adsorbed and thus increase the current flow when the device is exposed to UV light. The PD demonstrated a low dark current (~4 nA), which is crucial for minimising noise and improving the signal-to-noise ratio. This was achieved through the use of high-quality, ZnO NWs, which effectively suppress unwanted leakage current. The high R values are important, particularly for applications requiring the detection of low-intensity light. The demonstrated PD proves to be highly sensitive, with a minimum detection limit of 0.1 µW/cm^2^, making it suitable for a wide range of light detection applications.

Figure 3b shows the current ON/OFF ratio of PDs, which is an essential parameter for evaluating their sensitivity. It quantifies the PD’s ability to distinguish between the presence and absence of light by comparing the photogenerated current to the dark current. The PD exhibited a high current ON/OFF current ratio of >10^3^, which is primarily due to high photocurrent and low dark current values. Finally, the LDR of the PD is also calculated, which is a measure of the range of intensities as the responsivity is maintained at a constant, and is defined as^83^:

2 $${LDR}=20\,{\mathrm{log}}\left(\frac{{I}_{{Photo}}}{{I}_{{Dark}}}\right)$$

A higher LDR value indicates that the PD maintains a constant photoelectric conversion efficiency over a wide range of incident light intensities. The LDR of the PD at various illumination intensities and biasing potentials, presented in Fig. 3c, shows a maximum LDR of 80 dB. This extensive range demonstrates the PD’s ability to maintain consistent performance across a broad spectrum of incident light intensities, further highlighting its versatility and suitability for various applications.

To underline the exceptional performance of the fibre-based PD, Fig. 3d includes a schematic energy level diagram that presents a hypothesis to explain the observed results. The mechanism of UV sensing in PDs based on ZnO NWs has been extensively studied^84,85^. When PEDOT:PSS/Ag NWs (contact, p-type) and ZnO (photosensitive, n-type) are combined, electrons have the ability to migrate between the fibres of PEDOT:PSS/AgNWs and ZnO until they reach an equilibrium state. This migration process creates a depletion region at the interface of ZnO and PEDOT:PSS-Ag NWs, creating a potential difference at the junction. According to the photoelectric effect, when the PEDOT:PSS-Ag NWs/ZnO PD is exposed to UV light with a wavelength of 365 nm and photon energy of 3.396 eV, exceeding the ZnO bandgap, the electron-hole pairs are generated within the depletion region. The built-in electric field in the depletion layer, formed between p-PEDOT:PSS/Ag NWs and n-ZnO, enables the photogenerated electron-hole pairs to be efficiently separated and swept away from the depletion region, thus contributing to the generation of an external photocurrent. Furthermore, the generated hole reacts with the adsorbed oxygen ions on the surface, leading to the desorption of oxygen molecules and the release of the trapped free electrons on the NW surface. These events collectively decrease the depletion width and hence the current value of the fabricated ZnO/PEDOT:PSS-Ag NWs-based PD increases under UV illumination conditions^68^.

**Fig. 4:**
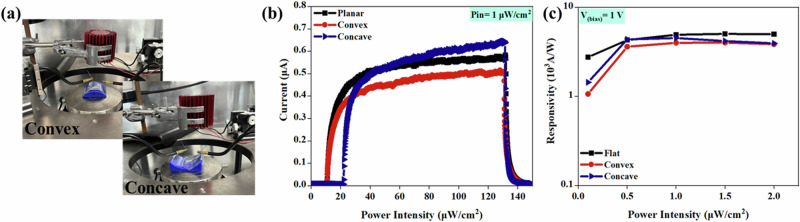
Bendability of the r-PD. **a** The experimental arrangement for electrical characterisation and photoresponses of the PDs on concave and convex conditions. **b** Current vs voltage plot at 1 V bias voltage and 1 μW/cm^2^. **c** Responsivity at 1 V bias voltage under different UV illumination intensities.

To quantify the mechanical flexibility, the PDs were characterised by mounting them on curved surfaces (concave and convex) with a 40 mm radius, as depicted in Fig. 4a. Figure 4b illustrates the time-domain photoresponse of the PD when exposed to a light intensity of 1 μW/cm^2^ at a bias voltage of 1 V, under concave, convex, and planar conditions. It is noticeable that the device mounted on the concave substrate exhibited a slightly higher photocurrent. This can be attributed to the focusing effect provided by the concave surface, which concentrates a higher amount of incident light onto the ZnO NWs. As a result, more photons are absorbed by the ZnO NWs, leading to a larger generation of electron-hole pairs and a higher photocurrent^86^. Another factor could be the reduced surface area available for the ZnO NWs to interact with the incident light on the concave substrate. On the other hand, when the PD is mounted on a convex substrate, the slightly increased surface area leads to a lower response. However, the changes in R under the different bending conditions, as shown in Fig. 4c, are insignificant, with slightly higher values observed for the planar substrate. This can be attributed to the better light coupling and distribution within the PD on the planar substrate. The absence of curvature or irregularities in the substrate surface reduces light scattering and promotes a more uniform distribution of incident light across the ZnO NWs, resulting in improved light absorption and, consequently, higher values of R. The ZnO NWs will be subjected to compressive and tensile strains when subjected to mechanical bending, resulting in a piezo potential generation that can assist in effective photocarrier separation. This piezo-phototronic effect could also contribute to the performance variation under mechanical bending^87^.

### Fibre-based PD with aligned ZnO NWs

In addition to the randomly oriented ZnO NWs based devices discussed above, we also investigated the PDs with aligned NWs. In this case, dielectrophoresis (DEP) method was used to orient the ZnO NWs across electrode. The PEDOT:PSS/Ag NWs nanofibers were used as the electrodes for DEP technique. As illustrated in Supplementary Figs. 2b and 3a, the ZnO NWs exhibited a well-defined alignment between the fibrous electrodes. The main concern was to preserve the performance of the fibrous electrodes as Schottky contacts despite the application of voltage and high frequency. To address this, a simple electrical test was conducted on the fibres before and after the DEP process. This analysis, detailed in Supplementary Fig. 3b, demonstrated that the alignment process of the NWs had no apparent impact on the electrical properties of the fibres, since the electrical resistance of the fibres before and after DEP remained approximately 40 Ω. These findings are important, as they underscore the ability of the conductive fibres to remain unaffected by high voltages and frequencies while fulfilling their role as electrodes in the PD device.

**Fig. 5:**
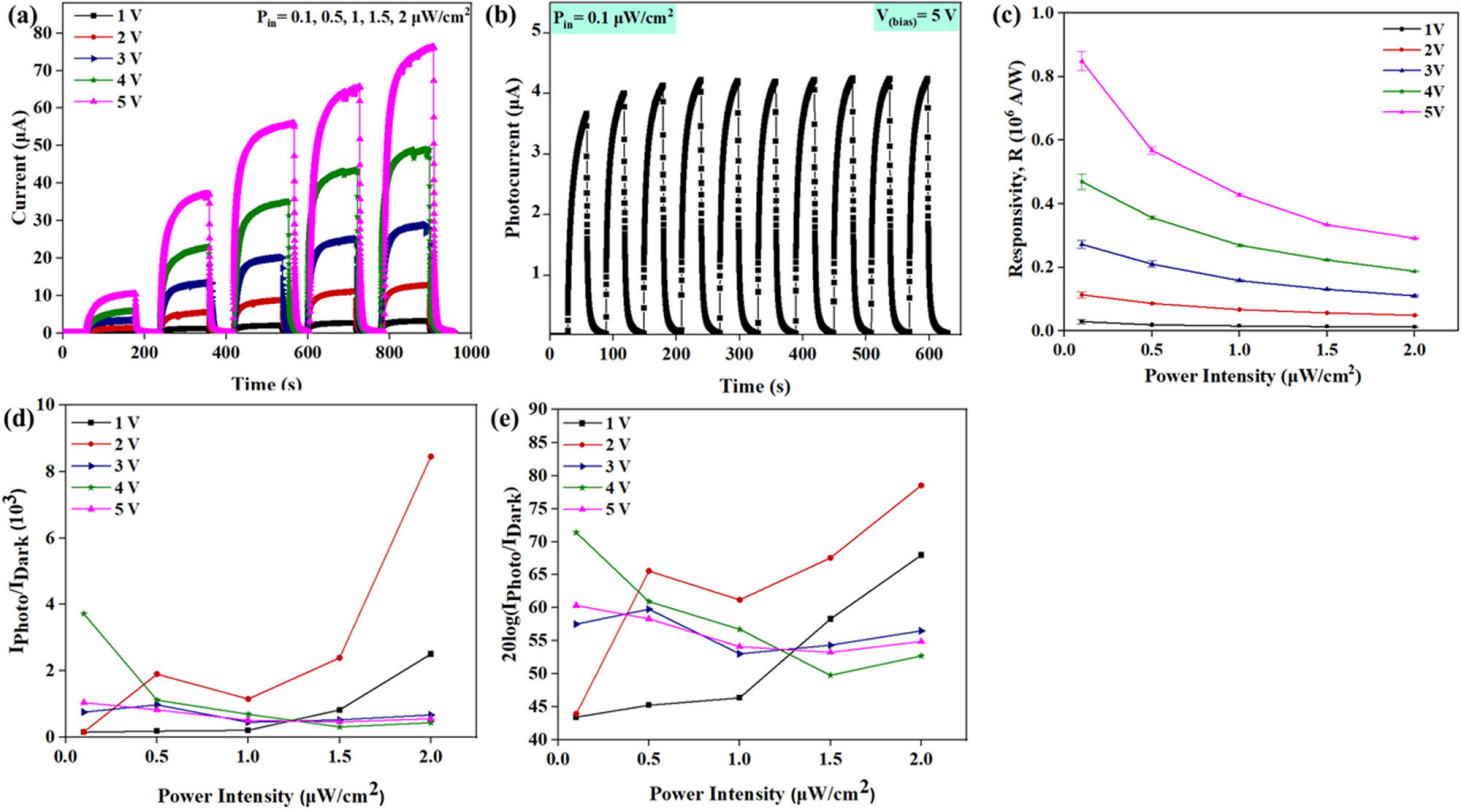
Electrical characterisation and performance of a-PD. **a** Light-illuminated temporal response characteristics at different bias voltages (1–5 V) under different UV illumination intensities. **b** Cyclic stability. **c** Responsivity. **d** Current ON/OFF ratio. **e** LDR plot under different illumination intensities.

The as-fabricated aligned NW PD (a-PD) underwent similar tests as the random NW PD (r-PD) under UV light with a wavelength of 365 nm. Figure 5a shows the photoresponse of the a-PD under different bias voltages (ranging from 1 to 5 V) and light illumination intensities from 0.1 to 2 μW/cm^2^. In all cases, the photocurrent demonstrated an upward trend with increasing incident UV light intensities. The peak photocurrent of 77.2 μA was observed at 2 μW/cm² light intensity and 5 V bias, while the lowest response of 0.5 μA was recorded at 0.1 μW/cm² light intensity and 1 V bias. The stability of the performance of the a-PD was assessed through cyclic exposure to 0.1 μW/cm² light, and the data are shown in Fig. 5b.

Using the photoresponses of the device under various light intensities and biasing conditions, the photodetection we extracted the figures of merit such as R, current ON/OFF ratio and LDR, as depicted in Fig. 5. Figure 5c illustrates the R under various exposed intensities and bias voltages. The highest values were observed at 5 V biasing under 0.1 µW/cm^2^ of UV light intensity, reaching 0.81 × 10^6 ^A/W.

Furthermore, a range of photodetection characteristics were extracted from the device’s photoresponses. These include crucial parameters such as R, current ON-OFF ratio, and LDR, all detailed in Fig. 5. The R is depicted in Fig. 5c, showcasing its highest values occurring under a 5 V bias and a UV light intensity of 0.1 μW/cm², peaking at 0.81 × 10^6^ A/W. Moreover, Fig. 5d presents the current ON/OFF ratio, while Fig. 5e displays the LDR. The LDR of the a-PD was calculated at 78 which is comparable with the LDR value of the randomly aligned ZnO NWs based PDs or r-PDs. In contrast, the R of the two devices has a noticeable difference, with the r-PD showing significantly higher values. This performance difference can be attributed to the different arrangement of ZnO NWs in the PDs (Supplementary Fig. 3). In r-PD, the disordered arrangement creates multiple paths for incident light to interact with the NWs – thus increasing the possibility of light absorption and electron-hole pair generation and leading to higher photoresponse. On other hand, in a-PD, the aligned arrangement of NWs may lower the light scattering and absorption due to the limited interaction points between the incident light and the aligned NWs. Another factor is NWs density. While aligned NWs improve charge transport in a-PDs, their lower density compared to r-PDs could reduce the electron-hole pair generation. This trade-off impacts the overall device performance and further investigated will be needed to firmly establish the boundary conditions. This will be further investigated in future works.

The performances of r-PD and a-PD were compared with the ZnO NWs-based state-of-the-art devices, as shown in Table 1. Previous ZnO NWs-based PDs have been manufactured using expensive and complex methods like photo-and nanoimprint lithography, and they have mainly been fabricated on rigid substrates. While some flexible devices have been developed recently, they often lack biodegradability and/or flexibility or exhibit lower photoresponsive characteristics. Differently from other works, here we present a promising alternative by utilising high-performance fibrous electrodes, resulting in flexible and biodegradable PDs with excellent characteristics. The r-PD demonstrates an R of 1.10 × 10^6^ A/W, and a photocurrent enhancement (I_Photo_/I_Dark_) of 81 × 10^3^, with a bias voltage of 5 V and a light intensity of 2 μW/cm^2^. Moreover, the a-PD exhibits an R of 0.29 × 10^6 ^A/W, and a photocurrent enhancement (I_Photo_/I_Dark_) of 0.55, for the same conditions.

**Table 1:** Comparative analysis of fibre-based PDs with state-of-the-art devices with ZnO NWs as the photosensitive element

Fabrication method	Electrode	Substrate	R [A/W]	I_Photo_/ I_Dark_	Flexible	Biodeg	Ref
Screen printing/spay coating	Ag	CS film	55	2.5 ×10^3^	Yes	Yes	^85^
Electron beam evaporation/drop casting-DEP	Au	PVC	4.3	10^3^	Yes	No	^86^
Drop casting on the mask/substrate	AgNW/Ag Paste	PDM S	54	5 ×10^5^	Yes	No	^20^
Extrusion printing/contact printing	Ag paste	PI tape	3.03 ×10^7^	~10^3^	No	No	^97^
Nanoimprint lithography	ITO	Glass	4.38 ×10^3^	3 ×10^3^	No	No	^98^
Rf sputtering/spray pyrolysis	ITO/Ag	Glass	0.25	0.08	No	No	^87^
Photolithography/RF magnetron sputtering	Au	SiO_2_/Si	14.9	−	No	No	^99^
Electrospinning/blade cutting/drop casting	PEDOT:PSS/ Ag NWs fibres	CA film	1.10 ×10^6^	81 ×10^3^	Yes	Yes	r-PD
Electrospinning/blade cutting/drop casting-DEP	PEDOT:PSS/ Ag NWs fibres	CA film	0.29 ×10^6^	0.55 ×10^3^	Yes	Yes	a-PD

### Optical transparency of the PDs

**Fig. 6:**
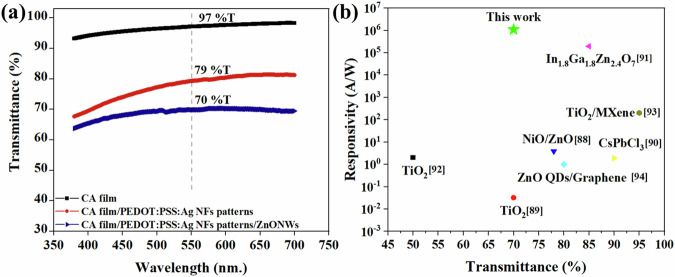
Transparency of the device. **a** Optical transmission spectra of the CA substrate, the CA substrate with the fibrous patterns and the device. **b** Performance comparison of the fibre-based PD of this work with other reported transparent PDs^88–94^, ZnO: zinc oxide, TiO_2_: titanium dioxide, NiO: nickel (II) oxide, CsPbCl_3_: cesium lead chloride, In_1.8_Ga_1.8_Zn_2.4_O_7_: indium gallium zinc oxide, QDs: quantum dots.

The optical transmittance of the different layers of PD, including the CA substrate, with the PEDOT:PSS-based fibrous patterns, and finally with the photosensitive material, was evaluated over a wavelength range of 380–700 nm, as presented in Fig. 6a. The pure CA substrate exhibited an optical transparency of 97% at a wavelength of 550 nm. With the addition of fibrous patterns, the optical transmittance decreased to approximately 79%, while the complete device, after incorporating ZnO NWs, showed a transmittance of 70% at the same wavelength. These results indicate that the device maintains an acceptable level of transparency and can potentially be integrated with other optoelectronic devices for applications, such as transparent image sensors, touchless interactive displays, and photo-skin for robots.

To reflect the performance level of the device, the key parameters, such as responsivity and transparency, were compared with previously reported transparent UV detectors using various photosensitive materials, including metal oxides, perovskites, 2D materials, and nanowires^88–94^, as shown in Fig. 6b. The fibre-based PD developed in this work exhibits excellent responsivity in comparison with other reported devices while maintaining acceptable optical transparency (>70%). Other studies using ZnO as the photosensitive material show similar transparency levels but have considerably lower responsivity than the current device. This enhanced performance may be attributed to the nature of the PEDOT:PSS/Ag NWs fibrous electrodes, which are easily bendable and conformable on the substrate. The use of fibre-based electrodes can reduce noise and interference, and improve the signal quality of photodetectors^53^. The extended, ultralong 1D structures inherent in fibres offer a significantly increased surface area, which further amplifies the sensitivity and efficiency^53^ of PDs. This significantly contributes to the enhanced performance of ZnO-based PDs, which makes them a promising solution for applications requiring both high responsivity and transparency.

### Degradability studies

**Fig. 7:**
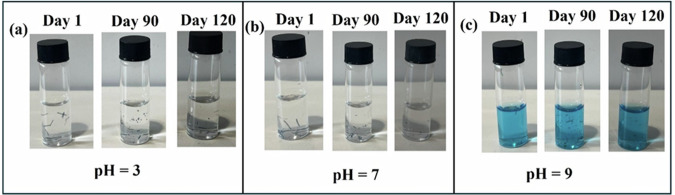
Sequential images collected at various stages of dissolution for biodegradable photodetector immersed in different PBS solutions at 37 °C. **a** pH = 3. **b** pH = 7. **c** pH = 11.

The photodetector consists of three layers made up of biodegradable moieties: CA as substrate, PEDOT:PSS@Ag as the conductive layer, and ZnO NWs as the photosensitive layer. Figure 7 represents the series of images taken during the dissolution process. Day 1 represents the image taken when the device was immersed in a buffer solution. It took approximately 24 weeks for the samples to be completely dissolved in the buffer solutions. Cellulose acetate dissolved completely after 4 weeks in all three buffer solutions. However, thread-like undissolved polymers of PEDOT:PSS@Ag were still present in the sample and took approximately six months to completely solubilize. Further, it was noticed that the rate of dissolution was faster in neutral and basic media and was slowest in acidic media. However, it must be noted that dissolution is not a true indicator of biodegradability. For instance, the degradability of CA largely depends on the degree of deacetylation^95^. Hence, it is difficult to degrade it to the desired level just by dissolution. Furthermore, PEDOT:PSS degrades very slowly with an estimated time period for its full degradation of 8+ years^96^. Thus, the estimation of byproducts formed is crucial in determining the actual rate of degradation. In light of this, the degradation studies of the as fabricated transient device are underway, and the complete analysis of the by-products will be carried out using liquid chromatography–mass spectrometry (LC-MS) analysis. An in-depth and systematic study will provide a comprehensive understanding of the plausible degradation mechanism of the presented transient device.

## Discussion

Flexible, transparent, and biodegradable photodetectors (PDs) have been developed using PEDOT:PSS/Ag NWs electrospun fibres as electrodes and ZnO NWs as photosensitive materials, in both random and aligned orientations. They showed excellent performance, with the PDs/random ZnO NWs achieving a a responsivity of 1.10 × 10^6^ A/W, while the PDs with aligned ZnO NWs achieved a a responsivity of 0.29 × 10^6^ A/W, under a 5 V bias and a UV light intensity of 2 μW/cm². The devices were also stable under dynamic exposure to UV light on flat, concave, and convex surfaces, making them an ideal candidate for various flexible and wearable applications. These PDs demonstrated an optical transparency of approximately 70% at a wavelength of 550 nm. In addition, the study shows that complete dissolution of the device within six months with faster dissolution in neutral and basic mediums opening new avenues towards fabricating sustainable electronic devices.

## Methods

### Materials

CA (Mn: 50 kDa), Ag NWs (0.5% w/w suspension in isopropyl alcohol (IPA)) with aspect ratio ~330 (diameter × length: 120–150 nm × 20 μm), Triton X-100, and dimethylformamide (DMF) were purchased from Sigma Aldrich. The ZnO NWs, A30 (diameter × length: 30–50 nm ×30 μm) were purchased from Novarials. PEDOT:PSS (PH 1000) was obtained from Ossila, and PEO (Mn: 100 kDa) was purchased from Alfa Aesar.

### Preparation of the substrate

A solution was prepared by mixing 1.6 wt. % of CA in 10 ml of DMF. The mixture was stirred continuously overnight until complete dissolution of CA. Subsequently, the CA solution was casted onto a glass petri dish and left at ambient conditions for 12 h, until the formation of a transparent film.

### Preparation of fibrous electrodes

The procedure for preparing the conductive fibres followed the steps explained in our previous work^57^. In brief, 0.225 g of polyethylene oxide (PEO) powder was mixed in 10 mL of PEDOT:PSS solution and stirred until it was dissolved. Then 1.3 mL of DMF and 20 μL of Triton X-100 were added to the solution and stirred for 2 h, and finally, 795 μL of Ag NWs dispersed in IPA solution was added and stirred for another hour until a uniform dispersion was obtained. The solution was then put into a syringe and placed onto the flow pump of the electrospinning setup (TL-PRO, TONGLI TL, Nanshan, Shenzhen, China), with a 10 cm distance from a rotating disc collector, covered with aluminium foil and CA films. A 19 G stainless steel needle was used to extract the conductive nanofibres and the collector was rotated at 1000 rpm. All experiments were conducted at room temperature and 30% humidity. After electrospinning, the fibres on the CA substrate were immersed in ethylene glycol (EG) for 10 mins and heated for 3 h at 90 °C.

### ZnO NW solution preparation

A ZnO NW solution was prepared by dispersing 2 mg of as-purchased ZnO NWs, with a diameter of approximately 30 nm and a length of around 30 μm, in 10 mL of IPA, followed by an hour of stirring at 200 rpm and 15 min of ultrasonication to achieve uniform dispersion.

### Device fabrication and characterisation

The fibrous conductive electrodes were patterned using Silhouette Cameo 2, a software-controlled plotter blade. For this, the CA film with PEDOT:PSS fibres were placed on a cutting mat for precise cutting. The blade length, force, and cutting speed were carefully adjusted to 0.5 mm, 19 gf, and 1 cm/s, respectively, to achieve a cut having a width of 37 ± 2 μm. The dimensions of the cut patterns were determined by using an optical microscope (Nikon ECLIPSE LV100ND) equipped with a digital camera (Nikon DS-Fi2). The microscope operation was controlled using LAS EZ software (Leica Application Suite, version 3.4.0, Leica Microsystems Switzerland Limited), and the images were captured at a magnification of 5x. The morphologies of the fibres and the devices were visualised using a JEOL IT 100 scanning electron microscope (SEM), with an accelerating voltage of 10 kV and imaged using InTouchScope software. Prior to characterisation, the samples were coated with a 15 nm thick gold film. The sheet resistance of the fibrous patterns was measured with Lucas/Signatone Four Point Probe, PrO4 series and a UV-2600 spectrophotometer (Shimazu Ltd) was used to assess their transparency. After that, 200 μl of the ZnO NWs solution were drop-casted in the active area equal to 0.01 mm^2^ to develop the device with randomly oriented NWs. The active area of the device (length (L) of 37 ± 0.42 μm and a width (W) of 356 ± 0.92 μm) was determined using the dimensions from the blade-cutting machine. Further, we used the dielectrophoresis (DEP) method to obtain devices with oriented ZnO. To this end, a 200 μl NW solution was drop casted onto the gap between the fibrous electrodes and an alternating current (AC) signal was applied using a signal generator (33120 A from Hewlett Packard) and an amplifier (A400DI from FLC electronics). The AC signal parameters were set to a peak-to-peak voltage (V_pp_) of 200 V and a frequency (f_AC_) of 1 MHz. During DEP, a non-uniform electric field is directed through the NWs solution to generate localised dipoles along the nanostructures, which results in the electric field gradient eventually drawing and aligning the NWs across the gap between the electrodes For the photo illumination experiments, a commercially available UV LED (ILH-x9090 UV LED board from Intelligent LED Solutions (ILS)) with a wavelength of 365 nm (λ = 365 nm) was used having a beam spot of diameter 6 cm. The light intensity of the LED was varied from 0.1 to 2 μW/cm^2^ for the PD characterisation by controlling the current supply using a current source and calibrated using a commercial photosensor. The IV and other electrical characterisation of the PDs were performed using a Keysight B2912A Semiconductor Device Parameter Analyser.

## Supplementary information

Supplementary file

Demonstration of reliable operation of transparent degradable photodetectors

## Data Availability

The data that support the findings of this study are available on request from the corresponding author.
