# Supplementary material for: Fully degradable, transparent, and flexible photodetectors using ZnO nanowires and PEDOT:PSS based nanofibres
Source: Npj Flex Electron. 2025 Mar 10;9(1):22. doi: 10.1038/s41528-025-00385-9 (PMC11893464; doi:10.1038/s41528-025-00385-9)
Supplement: Supplementary file 1 — Supplementary file [file 41528_2025_385_MOESM1_ESM.pdf]

Supplementary Information

Fully Degradable, Transparent, and Flexible Photodetectors using ZnO Nanowires and PEDOT:PSS based Nanofibres

Xenofon Karagiorgis[1,2], Nitheesh M. Nair[3], Sofia Sandhu[4], Abhishek Singh Dahiya[4], Peter J. Skabara[2] and Ravinder Dahiya[4]*

[1]School of Engineering, University of Glasgow, Glasgow, UK, G12 8QQ

[2] School of Chemistry, University of Glasgow, Glasgow, UK, G12 8QQ

[3]Institute of Smart Sensors, University of Stuttgart, Stuttgart, Germany, 70569

[4] Bendable Electronics and Sustainable Technologies (BEST) Group, Northeastern University, Boston, MA 02115, USA

*Corresponding author E-mail: r.dahiya@northeastern.edu

The PDF file includes:

Supplementary Figure 1. Bendability of the fibrous electrode. (a) Microscopic image of the patterned fibrous electrodes. The Inset image shows the morphology of the PEDOT:PSS/ AgNWs electrospun fibres, and an image of the CA substrate with the deposited PEDOT:PSS/AgNWs nanofibrous patterns. (b) Fibrous electrodes on CA film in a butterfly shape with LEDs in various bending radius.

Supplementary Figure 2. SEM images of the photodetector. (a) Device with random ZnO NWs. (b) Device with aligned ZnO NWs.

Supplementary Figure 3. Electrical properties of the fibrous electrodes (a) Microscopic image of the aligned ZnO NWs after DEP with the fibrous electrodes. (b) Electrical characterisation of the fibrous electrodes before and after DEP.

Supplementary Movie 1. The video shows the outstanding flexibility and durability of the developed PEDOT:PSS fibres under bending and twisting mechanical loadings.

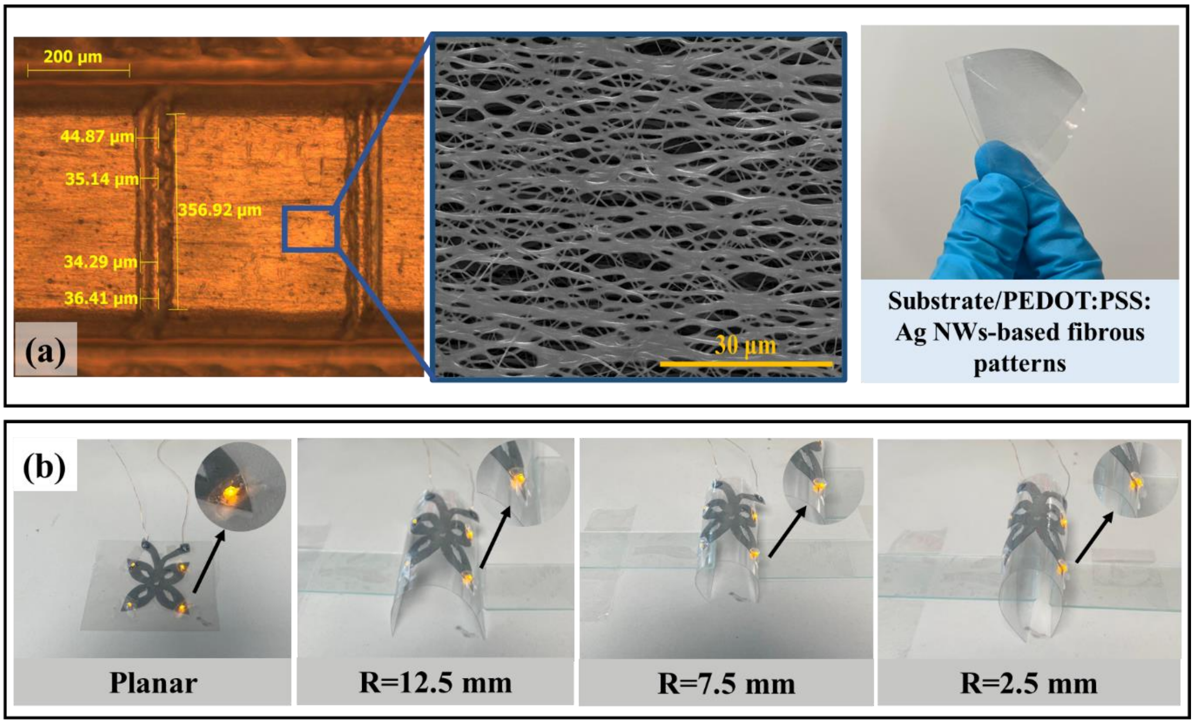

Supplementary Figure 1. Bendability of the fibrous electrode. (a) Microscopic image of the patterned fibrous electrodes. The Inset image shows the morphology of the PEDOT:PSS/ AgNWs electrospun fibres, and an image of the CA substrate with the deposited PEDOT:PSS/AgNWs nanofibrous patterns. (b) Fibrous electrodes on CA film in a butterfly shape with LEDs in various bending radius.

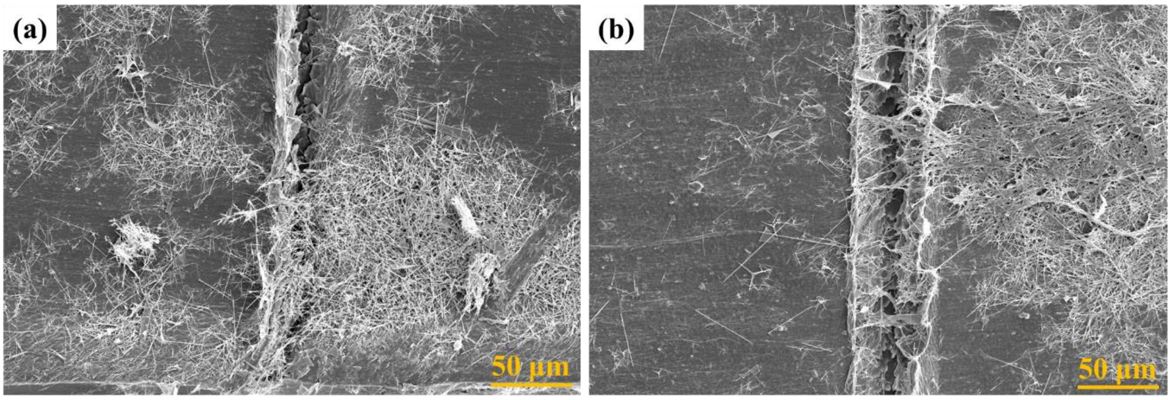

Supplementary Figure 2. SEM images of the photodetector. (a) Device with random ZnO NWs. (b) Device with aligned ZnO NWs.

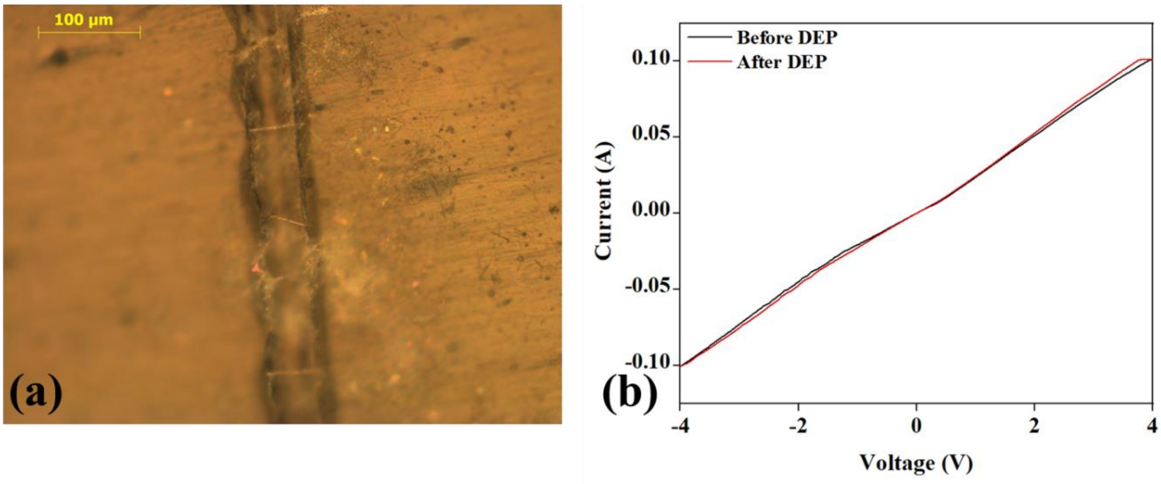

Supplementary Figure 3. Electrical properties of the fibrous electrodes (a) Microscopic image of the aligned ZnO NWs after DEP with the fibrous electrodes. (b) Electrical characterisation of the fibrous electrodes before and after DEP.
